# Very low-carbohydrate, high-fat, weight reduction diet decreases hepatic gene response to glucose in obese rats

**DOI:** 10.1186/s12986-018-0284-9

**Published:** 2018-07-31

**Authors:** Kathleen V. Axen, Marianna A. Harper, Yu Fu Kuo, Kenneth Axen

**Affiliations:** Department of Health and Nutrition Sciences, Brooklyn College, City University of New York, New York, USA

**Keywords:** Very low-carbohydrate diet, Weight loss, Insulin resistance, Lipogenesis, Gene expression, Obesity

## Abstract

**Background:**

Very low carbohydrate (VLC) diets are used to promote weight loss and improve insulin resistance (IR) in obesity. Since the high fat content of VLC diets may predispose to hepatic steatosis and hepatic insulin resistance, we investigated the effect of a VLC weight-reduction diet on measures of hepatic and whole body insulin resistance in obese rats.

**Methods:**

In Phase 1, adult male Sprague-Dawley rats were made obese by ad libitum consumption of a high-fat (HF1, 60% of energy) diet; control rats ate a lower-fat (LF, 15%) diet for 10 weeks. In Phase 2, obese rats were fed energy-restricted amounts of a VLC (5%C, 65%F), LC (19%C, 55%F) or HC (55%C, 15%F) diet for 8 weeks while HF2 rats continued the HF diet ad libitum. In Phase 3, VLC rats were switched to the HC diet for 1 week. At the end of each phase, measurements of body composition and metabolic parameters were obtained. Hepatic insulin resistance was assessed by comparing expression of insulin-regulated genes following an oral glucose load,that increased plasma insulin levels, with the expression observed in the feed-deprived state.

**Results:**

At the end of Phase 1, body weight, percent body fat, and hepatic lipid levels were greater in HF1 than LF rats (*p* < 0.05). At the end of Phase 2, percent body fat and intramuscular triglyceride decreased in LC and HC (*p* < 0.05), but not VLC rats, despite similar weight loss. VLC and HF2 rats had higher HOMA-IR and higher insulin at similar glucose levels following an *ip* glucose load than HC rats (*p* < 0.05). HC, but not VLC or HF2 rats, showed changes in *Srebf1, Scd1,* and *Cpt1a* expression (*p* < 0.05) in response to an oral glucose load. At the end of Phase 3, switching from the VLC to the HC diet mitigated differences in hepatic gene expression.

**Conclusion:**

When compared with a high-carbohydrate, low-fat diet that produced similar weight loss, a commonly used VLC diet failed to improve whole body insulin resistance; it also reduced insulin’s effect on hepatic gene expression, which may reflect the development of hepatic insulin resistance.

## Background

Very low carbohydrate (VLC, < 10% of energy) diets that are used to reduce body weight and improve glycemic control and insulin sensitivity in obese individuals [1, 2] generally provide > 50% of energy as fat [3–5]. High levels of dietary fat are implicated in the development of hepatic steatosis [6–8] which is prevalent in the obese population and is associated with hepatic insulin resistance and development of Type 2 diabetes mellitus [9]. Since obese individuals who are at risk for Type 2 diabetes mellitus may utilize a VLC diet to lose weight, it is important to understand the effect of VLC diets on hepatic response to insulin.

Although VLC diets have been used successfully for weight loss [10] and management of post-meal glycemia in humans [1, 11, 12], their impact on hepatic or whole body insulin resistance remains unclear [13]. Long-term, ad libitum consumption of a VLC diet by lean rats produced diabetes [14];in another study, consumption of a VLC diet by lean rats lowered their fasting blood glucose and insulin levels, but also produced glucose intolerance [15, 16], as well as hepatic and whole body insulin resistance when compared with an isocaloric high-carbohydrate, low-fat diet [15]. Previous studies from our laboratory in obese rats showed that an energy-restricted VLC diet produced less reduction in visceral fat, and hepatic and intramuscular lipid levels, and less improvement in glucose tolerance than an isocaloric high-carbohydrate, low-fat diet that yielded similar weight loss [17, 18]. The correlation between hepatic lipid concentration and glucose intolerance in that study [18] provided the impetus for the present investigation of the effects of a VLC weight-reduction diet on hepatic and whole body insulin resistance.

Ketosis diets, which are used to treat epilepsy in children [19], contain 0–10% carbohydrate (C), < 5% protein (P) and > 80% fat (F) [20]. Ketosis diets can increase percent body fat and hepatic triglyceride levels [21–23], and produce inflammation [23–25], liver damage [23, 24] and hepatic apoptosis [26] in rodents. Although ketosis diets lowered basal blood glucose and insulin levels in normal mice [27] and murine models of type 2 diabetes [23, 28], these diets also produced glucose intolerance, as well as hepatic [27] and whole body insulin resistance [29]. These results show that VLC diets with a low protein content can produce hepatic steatosis and hepatic insulin resistance.

Since the VLC diets typically used by obese individuals for weight loss have a moderate or high (≥ 25% of energy) protein content [3, 5, 30], the present study employed diets of different carbohydrate and fat, but of similarly high (26–30%) protein, contents. We compared the effects of a VLC diet on hepatic response to insulin, with that of a high-carbohydrate, low-fat diet (HC) that produced the same weight loss in dietary obese rats. Hepatic response was defined as the difference between expression of insulin-regulated genes after an oral glucose load, that raised plasma insulin levels, with expression of these genes in the feed-deprived state. We hypothesized that hepatic gene response to insulin would be impaired in rats on the VLC vs. HC diet, even with the same weight loss.

## Methods

### Research design

**Fig. 1:**
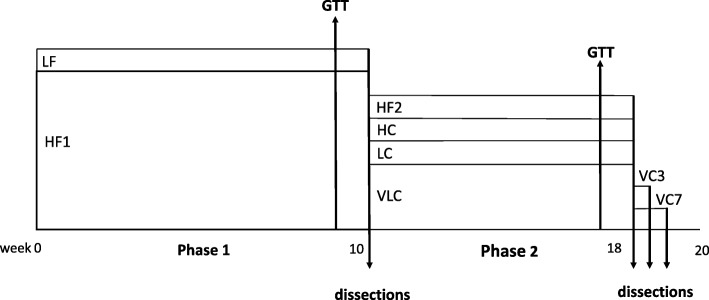
Experimental Design. GTT, intraperitoneal glucose tolerance test; LF, lower fat; HF, high fat; VLC, very low carbohydrate; LC, low carbohydrate; HC, high carbohydrate; VC, change from very low carbohydrate to high carbohydrate

In order to compare the effects of weight reduction by a VLC diet with that of a high-carbohydrate, lower-fat diet (HC), we first made rats obese in Phase 1 by ad libitum consumption of a high-fat diet for 10 weeks (HF1); rats consuming a lower-fat diet ad libitum served as a normal control group (LF, Fig. 1). In Phase 2 (8 weeks), obese rats from Phase 1 were given energy-restricted amounts of one of three diets, VLC, HC or LC, adjusted in amount to ensure similar weight reduction in all three groups. The LC diet was used to study the effects of a high-fat intake in the absence of extreme carbohydrate restriction (Table 1). A fourth group of rats (HF2) continued to feed ad libitum on the HF diet, to serve as an obese control. In Phase 3 (1 wk), some of the rats on the VLC diet in Phase 2 were switched to the HC diet for 3 or 7 days (VC3, VC7) in order to assess the persistence of the effects of the VLC diet after a switch to a high-carbohydrate, lower-fat diet. At the end of each of the three phases, metabolic and body composition analyses were performed, as well as measurement of hepatic gene expression both in the feed-deprived and post-glucose conditions (Fig. 1).

**Table 1:** Composition of experimental diets^b^

		Diet Group			
	LF	HF	VLC	LC	HC
Diet number^a^	D10112401	D10112402	D10112403	D10112406	D10112404
Ingredients (g/kg of diet)					
casein	282.0	321.9	393.8	313.0	274.8
L-cystine	4.2	4.8	5.9	4.6	4.1
corn starch	386.3	10.1	60.4	234.2	503.4
maltodextrin 10	117.5	157.2	0.0	0.0	0.0
sucrose	0.0	62.9	0.0	0.0	0.0
cellulose	94.0	62.9	67.7	61.1	91.6
soybean oil	15.0	72.9	90.0	70.9	14.4
lard	41.8	209.3	258.4	203.6	41.3
high oleic acid safflower oil	5.6	26.4	32.6	25.7	5.2
Total minerals	42.3	56.6	88.6	80.1	60.0
Vitamin mix V10001	9.4	12.6	0	0	0
Vitamin mix V10001C	0	0	2	1.8	1.4
Choline bitartrate	1.9	2.5	2.6	2.4	1.8
Cholesterol (mg/kg of diet)	86.5	215.1	264	209.2	84.7
Energy (kcal/g)	3.8	5.1	5.3	4.9	3.7
% of Energy					
Carbohydrate	55	19	5	19	55
Fat	15	55	65	55	15
Protein	30	26	30	26	30

^a^Research Diets, New Brunswick NJ

^b^*LF* lower-fat, *HF* high-fat, *VLC* very low-carbohydrate, *LC* low-carbohydrate, *HC* high-carbohydrate

### Animals and diets

Male Sprague-Dawley rats (Charles River Laboratories, MA) ~ 3 months of age (~ 290 g) were divided into two weight-matched groups*.* The control group (*N* = 8) consumed an LF diet (55%C, 15%F; Research Diets, NJ) (Table 1), similar in macronutrient composition to the standard AIN-76 diet [31]; the group to be rendered obese consumed an HF diet (19%C, 55%F) which contained 12% of kcal as sucrose in order to promote hyperphagia. Ad libitum feed intake, corrected for spillage, was measured during wk. 6; body weights were recorded twice a week. After 8 wk. of ad libitum HF feeding, body weight outliers were removed and HF rats (*N* = 58) were divided into five groups matched for both mean and range of body weights; one group of HF rats (HF1, *N* = 10) was tested for glucose tolerance and then dissected at wk. 10 (Fig. 1). During wk. 11–18, the remaining four groups of obese rats either continued to consume the HF diet ad libitum (HF2) or received daily restricted amounts of one of three diets (VLC, LC, or HC) that provided ~ 70% of the mean LF energy intake during Phase 1, adjusted as needed to maintain similar body weights among the groups. This level of energy restriction was used to induce a small weight loss in the growing rats. The VLC group (*N* = 24) received a 5%C, 65%F diet; the LC group (N = 8) a 19%C, 55%F diet; and the HC group (N = 8) a 55%C, 15%F diet. The LC and HC diets had similar macronutrient compositions to the HF and LF diets, respectively, but unlike those diets, they were consumed in hypocaloric amounts (Table 1). All diets had similar protein levels (26–30%), and were comprised of the same fat sources, resulting in the same distribution of polyunsaturated fat (36% PUFA) and monounsaturated fat (38% MUFA) among diets. None of the three weight-reduction diets contained sucrose. The VLC group included more rats than the other groups because VLC rats were later divided into 3 weight-matched groups (each *N* = 8): one group for dissection at wk. 18–19 and two groups for Phase 3 of the study (Fig. 1). The VLC, LC and HC rats received fresh rations daily; their feed intakes were measured for three consecutive days per week during weeks 13 and 16 of the study. To investigate the reversibility of the effects of the VLC diet, the VLC rats remaining after wk. 18 were switched to the HC diet for three (*N* = 8, VC3) or seven (*N* = 8, VC7) days (Phase 3). Rats were housed at 22^o^ C, on a 12 h:12 h light-dark cycle; all procedures were approved by the Brooklyn College Institutional Animal Care and Use Committee (Protocol 248).

### Metabolic profile

At wk. 9, HF1 (N = 10) and LF (N = 8) rats were feed deprived overnight (16 h) and a glucose tolerance test (GTT) was administered by intraperitoneal (*ip*) injection of 50% glucose (1 g/kg of body weight); glucose was measured in tail blood samples taken before (*t* = 0) and at 10, 20, 30, 45 and 75 min after the glucose load. Similarly, at wk. 17 an *ip* GTT, along with measurement of feed-deprived plasma ketone levels, was performed following overnight feed deprivation on 6 rats from each of the four diet groups in Phase 2; rats from the energy-restricted groups were chosen to match body weights: VLC (640 ± 13 g, mean ± SEM), LC (635 ± 8), and HC (631 ± 14).

### Tissue collection

At the end of Phase 1, 8 LF and 10 HF1 rats were feed deprived overnight (16–19 h); half of the rats in each diet group were dissected in the feed-deprived state and half 3 h after an oral load of 50% glucose administered by gavage (2 g/kg). The effect of the oral glucose load on plasma glucose and insulin levels was measured at 30 min intervals (0–90 min) in a separate group of 5 LF and 6 HF rats, confirming that glucose and insulin levels remained elevated between 30 and 90 min in both groups; measurements were made in these other rats to avoid the stress of blood draws before tissue sampling. In all phases of the study, the order of diet groups and conditions (feed-deprived vs. post-glucose) were systematically counterbalanced to ensure that they did not differ among groups. In Phases 2 and 3, all rats to be dissected were feed deprived overnight and tissue samples were obtained from each diet group in the feed-deprived state (*N* = 4) or 3 h after the first of two doses of glucose (each 2 g/kg), given by gavage 90 min apart (*N* = 4); a higher glucose load than that used in Phase 1 was employed to provide a stronger stimulus for insulin release. The VC3 and VC7 groups were similarly treated and dissected after three or seven days after switching to the HC diet, respectively.

Rats were anesthetized by *ip* injection of Na pentobarbital (55 mg/kg, Sigma-Aldrich). Liver samples were freeze clamped and placed in liquid Nitrogen for later measurement of lipid concentration, or stored in RNAlater (Qiagen) for later extraction of nucleic acids. Blood (~ 10 mL) was drawn from the descending aorta into a heparinized syringe (10 USP units/ mL of blood, Schein); plasma was frozen for later measurement of insulin, leptin, and triglyceride. Fat pads were dissected from the epididymal, omental-mesenteric, retroperitoneal and entire subcutaneous depots, and covered in plastic until weights were recorded. Rats died under anesthesia following the blood draw.

### Blood and tissue analysis

Triglyceride concentration in plasma and in muscle lipid extracts was measured using a kit (Wako), as was plasma concentration of β-hydroxybutyrate (Stanbio Laboratory). Blood glucose concentration was measured by glucometer (One Touch, LifeScan); plasma insulin and leptin concentrations were measured by radioimmunoassay (Millipore).

Total lipid was extracted from homogenized liver samples [32], and from muscle fibers dissected from lyophilized soleus samples [33]; triglyceride from the muscle lipid extract was solubilized in isopropanol for assay as above.

### RNA extraction and quantitative real-time PCR analysis

Total liver RNA was isolated using the RNeasy mini kit (Qiagen), and reverse transcribed using the High Capacity cDNA Reverse Transcription Kit (Applied Biosystems). Gene expression was measured using TaqMan expression assays and master mix (Life Technologies) under conditions specified for the product; two assays were performed for each sample for every gene of interest (CFX Connect, BioRad). Each PCR run included triplicates of cDNA (5 ng total cDNA) for each gene and a no-template control, as well as a calibrator from pooled samples in that phase of the study in order to document inter-run comparability for all genes. Relative expression was normalized for transcript levels of the reference gene, ribosomal protein P2 (Rplp2), whose expression was unaffected by diet or feed-deprived vs. post-glucose conditions.

The effect of diet on hepatic insulin resistance was assessed by comparing post-glucose with feed-deprived expression of genes known to be regulated by insulin. SREBP1c (Sterol Regulatory Element Binding Protein), a major regulator of lipogenesis whose gene (*Srebf1*) transcription is increased by insulin, is attached to the endoplasmic reticulum via the protein Insig2 (Insulin-induced gene 2); once released, the mature form of SREBP1c is transported to the nucleus where it binds to regulatory elements of its target genes, including *Acaca* (Acetyl-CoA Carboxylase), *Fasn* (Fatty Acid Synthase), *Scd* (Stearoyl-CoA Desaturase1), and *Gck* (Glucokinase). Insulin decreases the expression of *Insig2*, thereby promoting the action of SREBP1c. Insulin also decreases expression of *Pck1* (Phosphoenolpyruvate Carboxykinase), and *Cpt1a* (Carnitine Palmitoyl Transferase 1).

### Statistical analysis

Data are shown as means ± SEM. The area, above feed-deprived levels, under the glucose vs. time curve (AUC) for the *ip* glucose tolerance test was calculated as follows:

$$ \mathrm{AUC}=-55\ {\mathrm{G}}_0+10\ {\mathrm{G}}_{10}+10\ {\mathrm{G}}_{20}+12.5\ {\mathrm{G}}_{30}+15\ {\mathrm{G}}_{45}+7.5\ {\mathrm{G}}_{75} $$

Where G represents the blood glucose concentration and the subscript represents the sampling time in minutes after the glucose load.

Analyses were performed using SPSS version 24 software. Comparisons between diet groups and between conditions were analyzed by Analysis of Variance and post hoc Bonferroni analyses; body weight changes within subjects were analyzed by repeated measures one-way ANOVA. For PCR results, data for each phase were analyzed both as 2 ^–dCt^ and log-transformed as dCt; significant effects were essentially the same for both methods, but because variances among diet groups were unequal for several genes using 2^-dCt^ but not using dCt, significance is reported based on dCt values. Differences with values of *p* < 0.05 were considered to be significant.

## Results

### Food intake and body composition

Ad libitum energy intake, measured at wk. 6 of Phase 1, was greater in HF1 vs. LF rats (106 ± 2 vs. 94 ± 3 kcal/day, *p* < 0.001). By the end of Phase 1 (wk 10), body weights, visceral fat, total body fat (sum of dissected fat), % body fat (total body fat × 100%/ body weight) (*p* < 0.01), hepatic lipid levels (*p* < 0.05, Table 2) and plasma leptin levels (*p* < 0.001) were greater in HF1 vs. LF rats, demonstrating that HF1 rats were obese and had hepatic steatosis (> 5% of liver weight as lipid) at the end of Phase 1. Soleus intramuscular triglyceride (TG) levels did not differ between HF1 and LF rats (8.1 ± 0.4 vs. 5.9 ± 1.7 mg/g).

**Table 2:** Effects of diet on body composition in male rats^1^

DIET	Body	Visceral	Total	Body	Liver	Muscle	Leptin
	Weight (g)	Fat (g)	Fat (g)	Fat (%)	Lipid (mg/g)	TG (mg/g)	ng/mL
Phase 1							
LF	558 ± 24^b^	39 ± 5.1^b^	67.2 ± 8.3^c^	11.3 ± 1.2^b^	49.9 ± 5.8^c^	5.9 ± 1.7^bcd^	11.6 ± 2.1^b^
HF1	665 ± 32^a^	69.0 ± 11.1^a^	130 ± 17.3^ab^	19.1 ± 1.9^a^	73 ± 5.2^a^	8.1 ± 0.4^ab^	42.6 ± 8.2^a^
Phase 2							
HF2	748 ± 13^a^	78.2 ± 7.2^a^	148 ± 9.6^a^	19.7 ± 1.0^a^	72.1 ± 6.9^a^	9.5 ± 0.7^a^	38.9 ± 5.9^a^
VLC	628 ± 14^b^	51.1 ± 3.7^ab^	92.9 ± 7.8^bc^	14.7 ± 1^ab^	52.5 ± 6^bc^	6.9 ± 1.4^bc^	14.1 ± 2.2^b^
LC	613 ± 0.9^b^	42.2 ± 3.6^b^	75.6 ± 5.4^c^	12.3 ± 0.8^b^	67.8 ± 4.9^ab^	5.5 ± 0.7^cd^	8.7 ± 1.1^b^
HC	610 ± 11^b^	37.9 ± 2.5^b^	72.5 ± 6.1^c^	11.9 ± 1^b^	53 ± 6.9^bc^	4.5 ± 0.7^cd^	7.9 ± 1.0^b^
Phase 3							
VC3	632 ± 10^b^				60.8 ± 4.2^bc^	3.4 ± 0.1^e^	8.6 ± 1.8^b^
VC7	644 ± 11^b^				54.1 ± 3.5^bc^	4.9 ± 1.1^cd^	7.4 ± 1.4^b^

^1^Values are means ± SEM. Labeled means in a column without a common letter differ, *P* < 0.05; the letter a represents the highest value .HOMA-IR units are (mmol/L)(μU/mL)/22.5. LF, lower-fat; HF1, high-fat Phase 1; HF2, high-fat Phase 2; VLC, very low-carbohydrate; LC, low-carbohydrate; HC, high-carbohydrate

Calculated energy intakes were similar at wk. 13 vs.16 in the three energy-restricted groups; intakes at those times were lower in LC (63 ± 2 kcal/d) than VLC (69 ± 1) or HC (68 ± 0.3) groups (*p* < 0.01). As intended, these restricted daily energy intakes were lower than those of the LF (94 ± 3) or HF1 (106 ± 2 kcal/day) groups in Phase 1 (*p*< 0.001). Body weights did not differ among the energy-restricted groups throughout wk. 11–18, and all three groups weighed less than the HF2 group throughout wk. 13–18 (*p* < 0.001). Although visceral, total and % body fat (*p* < 0.001), as well as soleus intramuscular TG (*p* < 0.05), decreased in LC and HC groups from that in the HF1 group at the end of Phase 1 (Table 2), no significant reduction in these measures was observed in the VLC group. Body weight and visceral, total and % body fat did not change in HF2 rats between wk. 10 and 18. Plasma leptin concentration was higher in HF1 (wk 10) and HF2 (wk 18) than in VLC, LC or HC groups at wk. 18 (*p*< 0.0001). HF1 and HF2 rats had the same level of hepatic lipid, while rats on the VLC and HC, but not LC, diets decreased hepatic lipid concentration during Phase 2 (*p* < 0.01).

Total weight loss after the switch from the VLC to the HC diet in Phase 3 did not differ between rats on the HC diet for 3 days (VC3: 30 ± 2 g) or 7 days (VC7: 25 ± 5 g). Like VLC rats, body weights of VC3 and VC7 rats remained lower than that of HF2 rats, and concentrations of hepatic lipid and plasma levels of leptin (Table 2) were lower than those of HF1 or HF2 rats (*p* < 0.05). In contrast, the switch from the VLC to the HC diet resulted in lower intramuscular TG levels in VC3 and VC7 than in HF1 or HF2 rats. These results support the lower effectiveness of the VLC vs. the HC diet in reducing muscle lipid during weight loss.

### Metabolic profile

Feed-deprived values for plasma glucose and insulin concentrations did not differ between LF and HF1 rats at wk. 6, nor did glucose tolerance differ, as assessed by the area under the glucose vs. time curve (AUC) during the *ip* GTT (Table 3).

**Table 3:** Dietary Effects on Plasma Levels of Glucose, Insulin and Ketones in Rats^1^

DIET	HOMA-IR	Glucose	ipGTT AUC	ipGTT insulin		β-hydroxy butyrate
		mmol/L	(mM)(min)	ng/mL		mmol/L
		t0		t0	t30	
Phase 1						
LF	10.9+1.2^a^	5.5+0.2	542+41^ab^	1.58+0.12^ab^		
HF1	12.7+1.8^a^	5.3+0.1	674+38^a^	1.85+0.22^a^		
Phase 2						
HF2	7.8+3.2^a^	5.9+0.4	453+53^ab^	1.03+0.41^bc^	1.97+0.49^a^*	0.62+2.30
VLC	10.0+2.3^a^	6.5+0.5	442+22^b^	1.25+0.29^b^	2.5+0.61^a^*	0.5+0.07
LC	3.3+1.0^b^	6.2+0.1	482+52^ab^	0.41+0.13^cd^	1.69+0.20^ab^*	0.49+0.05
HC	1.3+0.1^b^	5.7+0.5	442+50^b^	0.18+0.02^d^	0.52+0.13^b^	0.51+0.16

^1^Values are means + SEM. Labeled means in a column without a common letter differ, P<0.05. * Different from t0, P<0.05. HOMA-IR units are (mmol/L)(μU/mL)/22.5. LF, lower- fat; HF1, high-fat Phase 1; HF2, high-fat Phase 2; VLC, very low-carbohydrate; LC, low-carbohydrate; HC, high-carbohydrate

**Fig. 2:**
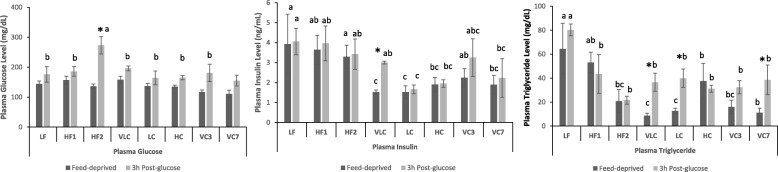
Effect of diet on plasma glucose, insulin and triglyceride levels in rats at dissection. Rats were feed-deprived for 16–19 h; half of the rats were then given an oral load of 50% glucose. Plasma samples were obtained from anesthetized animals either in the feed-deprived state or 3 h post glucose. Values are means ± SEM. Labeled means for a given diet group for a particular condition (feed-deprived or post-glucose) without a common letter differ, *p* < 0.05. * Different from Feed Deprived, *p*< 0.05. LF, lower-fat; HF1, high-fat Phase 1; HF2, high-fat Phase 2; VLC, very low-carbohydrate; LC, low-carbohydrate; HC, high-carbohydrate; VC3, switched from VLC to HC 3 days; VC7, switched from VLC to HC 7 days

At the time of dissection, plasma levels of glucose and insulin did not differ between LF and HF1 rats in either the feed-deprived or post-oral glucose conditions (Fig. 2). Plasma triglyceride (TG) levels were higher in the LF vs. the HF1 group (*p* < 0.05).

Feed-deprived levels of plasma glucose did not differ among any groups (*t* = 0, *ip*GTT, Table 3); the higher corresponding levels of insulin in LF, HF1, HF2, and VLC groups vs. the HC group resulted in higher HOMA-IR values in the LF, HF1, HF2 and VLC groups vs. the HC group (*p* < 0.05). Similarly, higher feed-deprived levels of insulin in the HF1 group resulted in higher HOMA-IR values in the HF1 than the LC group (Table 3, *p* < 0.05). These data provide evidence for whole body insulin resistance in the HF1 rats before weight reduction, and in the VLC rats after weight reduction. The *ip* glucose load resulted in similar AUC values in all groups in Phase 2 (Table 3). Although both VLC and HC groups showed a reduction from the HF1 group’s AUC values (*p* < 0.05), and glucose values of VLC and HC rats did not differ at any time point, the VLC group had a higher insulin concentration at 30 min post-injection than the HC group (*p* < 0.01); the higher plasma insulin for the same glucose level in VLC vs. HC rats provides evidence for whole body insulin resistance in VLC rats. Plasma β-hydroxybutyrate levels, measured after an overnight fast during wk. 17, were in the ketotic range (> 0.5 mmol/L) for all Phase 2 groups and did not differ among the four diet groups; these levels of β-hydroxybutyrate exceeded those we previously reported for VLC or HC rats in the fed state [18], demonstrating that prolonged feed-deprivation can raise plasma ketone levels in rats, even if they consume a high-carbohydrate diet.

In blood samples obtained from anesthetized rats during dissection, feed-deprived glucose levels did not differ among groups, but only HF1 rats still showed an elevation in glucose at 3 h after the oral glucose load (*p* < 0.001, Fig. 2). However, VLC rats had higher than feed-deprived insulin levels at 3 h after the oral load (*p* < 0.05); such persistent elevation in insulin levels is consistent with insulin resistance in VLC rats. Feed-deprived levels of plasma triglyceride were elevated in the HC group (HC > VLC ~ LC ~ HF2, *p* < 0.05) and remained high after the oral glucose load. The increase in plasma TG levels by the glucose load (*p* < 0.02) in both VLC and LC groups is consistent with insulin resistance.

*Phase 3: Switch from VLC to HC Diet* At the time of Phase 3 dissection, plasma insulin levels were higher (*p* < 0.05) in feed-deprived VC3 than they had been in VLC or LC rats at wk. 18, but this difference was no longer significant by day 7 after the switch to the HC diet (VC7 group).

### Hepatic gene expression

**Fig. 3:**
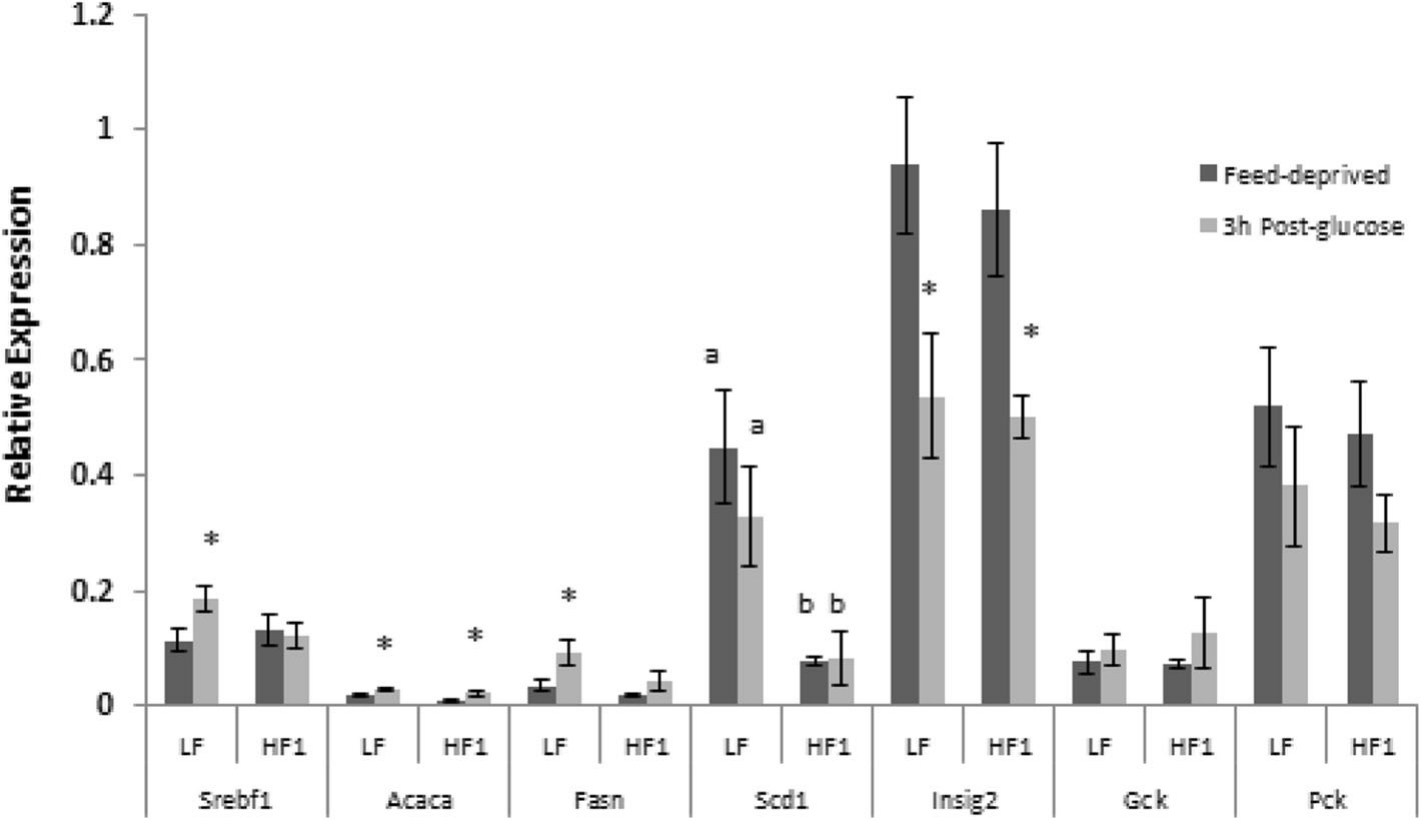
Effect of glucose on gene expression in rats consuming LF vs. HF1 diets. Liver samples were obtained from rats consuming either an LF (*N* = 8) or an HF (*N* = 10) ad libitum for ten weeks (Phase 1) feed-deprived 16 h (dark bars) and 3 h post-glucose (light bars). Relative hepatic gene expression is plotted as 2^-dCt^. Means ± SEM are shown; labelled means, for a given gene and condition, without a common letter differ from each other, *p* < 0.05. *Different from Feed-deprived, *p* < 0.05. *Acaca*: acetyl-CoA carboxylase; *Fasn*: fatty acid synthase; *Gck*: glucokinase; *Insig2*: insulin signaling protein 2; *Pck1*: phosphoenolpyruvate carboxykinase; *Scd1*: stearoyl-CoA desaturase-1; *Srebf1*: Sterol regulatory element-binding protein 1. LF, lower-fat; HF1 high-fat Phase 1

The effects of the HF diet on regulation of hepatic gene expression by insulin was assessed by comparing levels of mRNA, transcribed from insulin-regulated genes, in liver samples obtained from rats in the feed-deprived state *vs.* those obtained at 3 h after an oral glucose load, which was used to raise plasma insulin levels. At the end of Phase 1, the glucose load increased the expression of *Srebf*, whose product SREBP1c is a major regulator of lipogenesis, as well as its target *Fasn,* in the LF but not the HF1 group (*p* < 0.05, Fig. 3). In response to glucose, HF1 and LF groups both showed increased expression of *Acaca* (target of SREBP1c) and decreased expression of *Insig2*. Expression of *Scd1* (target of SREBP1c), was higher in LF vs. HF1 rats in both the feed-deprived and 3 h post- oral glucose conditions (*p* < 0.01).

**Fig. 4:**
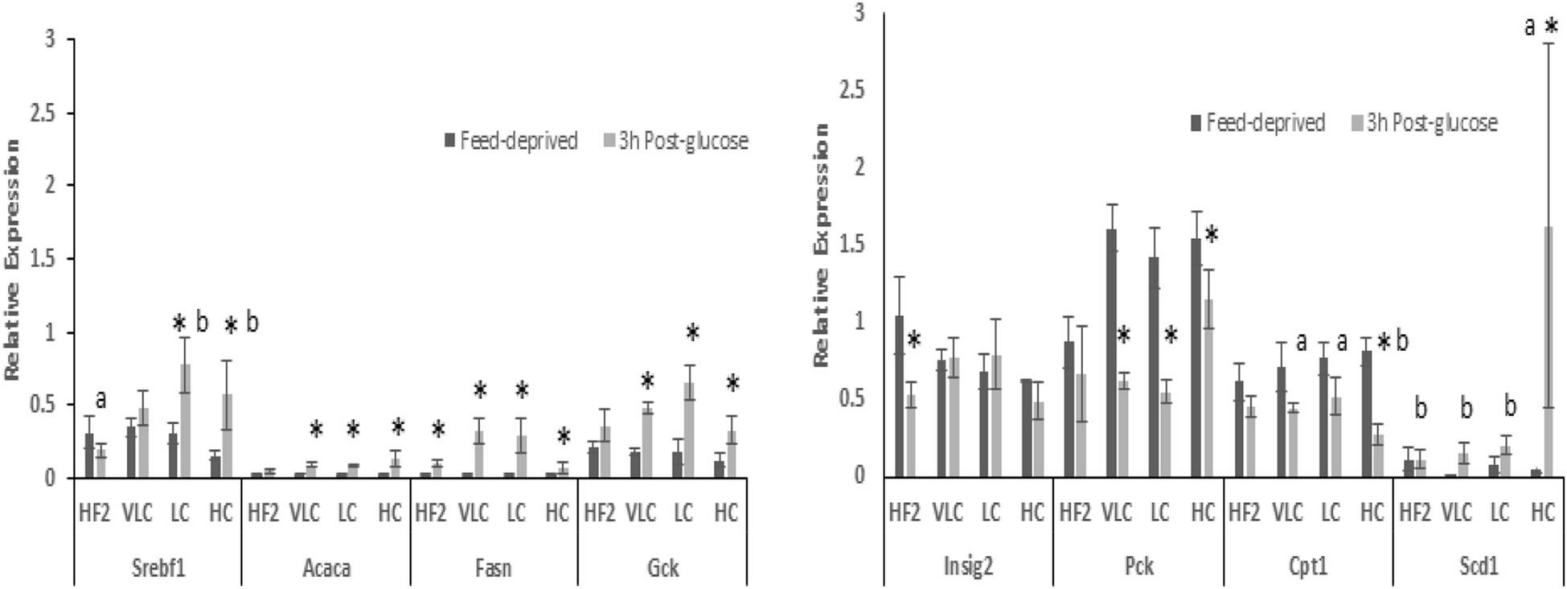
Effect of glucose on gene expression in rats consuming HF2 vs. VLC, LC or HC diets. Liver samples were obtained from obese rats continuing to consume an HF diet ad libitum or VLC, LC or HC diets in restricted amounts for 8 weeks (Phase 2); feed-deprived 16h (dark bars) and 3 h post-glucose (light bars). Relative hepatic gene expression is plotted as 2^-dCt^. Means ± SEM are shown; labelled means, for a given gene and condition, without a common letter differ from each other, *p* < 0.05. *Different from Feed-deprived, *p*< 0.05. Ct ranges across all groups and conditions are shown in Parentheses; *Acaca*: acetyl-CoA carboxylase (28–32); Cpt1a: carnitine Palmitoyl transferase-1a (24–27); *Fasn*: fatty acid synthase (25–32); *Gck*: glucokinase (24–30); *Insig2*: insulin signaling protein-2 (24–27); *Pck1*: phosphoenolpyruvate carboxykinase-1 (21–27); *Scd1*: stearoyl-CoA desaturase-1 (24–32); *Srebf1*: Sterol regulatory element-binding protein(25–29); *Rplp2:*Ribosomal protein P2 (24–25); HF2, high-fat Phase 2; VLC, very low-carbohydrate; LC, low-carbohydrate; HC, high-carbohydrate

At the end of Phase 2, all groups increased *Acaca* mRNA after the glucose load (Fig. 4); VLC, LC and HC, but not HF2, rats showed effects of the load on expression of *Fasn* and *Gck* (increased) or *Pck1* (decreased). Only the HF1 group showed the expected decrease in *Insig2* mRNA in response to the glucose load. The glucose load increased expression of *Srebf1* in HC and LC, but not VLC or HF2 groups, while stimulation of *Scd1* and inhibition of *Cpt1a* expression were seen only in the HC group.

**Fig. 5:**
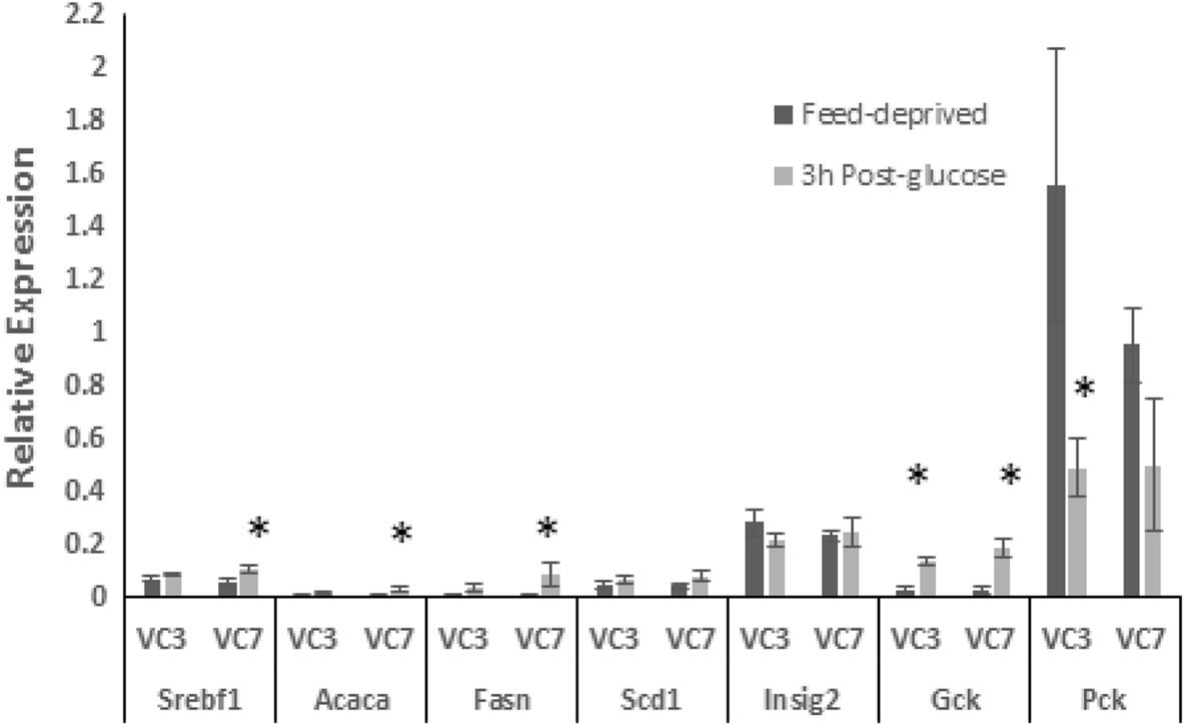
Effect of glucose on gene expression in rats switched from VLC to an HC diet. Liver samples were obtained from obese rats that had consumed restricted amounts of a VLC diet for 8 weeks and then were switched to isocaloric amounts of the HC diet for 3 (VC3) or 7 (VC7) days (Phase 3); 16 h feed-deprived (dark bars) and 3 h post-glucose (light bars). Relative hepatic gene expression is plotted as 2^-dCt^. Means ± SEM are shown. *Different from Feed-deprived, *P* < 0.05, ** *P* < 0.01, *** *P* < 0.001. *Acaca*: acetyl-CoA carboxylase; *Fasn*: fatty acid synthase; *Gck*: glucokinase; *Insig2*: insulin signaling protein-2; *Pck1*: phosphoenolpyruvate carboxykinase-1; *Scd1*: stearoyl-CoA desaturase-1; *Srebf1*: Sterol regulatory element-binding protein-1

In Phase 3, the VC3 group failed to increase *Srebf, Acaca,* or *Fasn* mRNA in response to the glucose load (Fig. 5), but after 7 days on the HC diet, VC7 rats showed responses qualitatively similar to those of HC rats for these genes, although neither group showed the increase in levels of *Scd1* mRNA exhibited by the HC group. Only VC3 rats decreased expression of *Pck1* after the glucose load; this may reflect the trend toward higher feed-deprived *Pck1* mRNA in VC3 vs. VC7 groups. In VC3 and VC7 rats, overall levels of mRNA for *Srebf1*, *Insig2*, *Acaca* and *Gck* were lower (*p* < 0.05) than those in VLC or HC rats at end of Phase 2, even when mRNA levels were analyzed with reference to a calibrator comprised of pooled samples from the respective phases (ddCt).

## Discussion

Ad libitum consumption of the HF diet for 10 wk. produced obesity characterized by increased body weight, % body fat, and plasma leptin levels as compared with the LF control group, as well as hepatic steatosis. Continued ad libitum consumption of the HF diet for another 8 weeks in Phase 2 did not change any of these measures of obesity. Eight weeks of energy restriction of obese HF2 rats on VLC, LC or HC diets produced similar reductions in body weights and plasma leptin levels.

In rats on the LC and HC, but not VLC, diets visceral fat, % body fat, and intramuscular TG were significantly reduced from that at wk. 10 (end of Phase 1), in agreement with our previous findings using VLC and HC diets [18]. In contrast, hepatic lipid levels decreased from HF1 values in the VLC and HC but not the LC group; this finding suggests that high dietary fat, coupled with an adequate amount of carbohydrate, promoted hepatic fat storage even during weight loss. These results show that the fat and carbohydrate compositions of the diets consumed in hypocaloric amounts during Phase 2 had differential effects on adipose tissue and hepatic fat loss during energy restriction.

Evidence for whole body insulin resistance in the VLC and HF 2 rats was observed during Phase 2 of the study. Higher HOMA-IR values in unanesthetized VLC and HF2 rats were due to elevated feed-deprived plasma insulin levels vs. those of HC rats. In addition, insulin levels were higher in VLC than HC rats at 30 min after the *ip* glucose load, despite similar plasma glucose AUC values. These results suggest that insulin release may have increased as an adaptation to tissue insulin resistance in VLC rats during Phase 2. In our previous study, the elevated plasma glucose levels after an *ip* glucose load in VLC vs. HC rats, but similar insulin levels, indicated insulin resistance [18]. The higher levels of plasma triglyceride in LF and HC vs. VLC groups are consistent with results reported in humans and rodents on high carbohydrate diets [34–36], and have been associated with hepatic lipogenesis [34].

The present study was designed to permit interpretation of differences in hepatic gene expression among diet groups to represent chronic adjustments, and not acute responses to the nutrient content of the current meal. To this end, all rats were feed-deprived for > 16 h before sampling, the same glucose stimulus was used for all groups in a given Phase, and each diet group’s gene expression responses to the oral glucose load were compared with that in the feed-deprived state.

The effects of diet composition on hepatic insulin resistance were evaluated by examining responses of insulin-regulated genes, many of which have roles in hepatic lipogenesis (*Srebf1*, *Insig2*, *Acaca*, *Fasn*, *Scd1*, *and Gck*). Although the lipogenic pathway (including de novo synthesis of fatty acids, DNL) is stimulated by insulin, there is disagreement concerning whether regulation of DNL is impaired by hepatic insulin resistance. In hepatic insulin resistance, insulin-regulated pathways that involve glucose metabolism are dysregulated (e.g., insulin fails to inhibit gluconeogenesis), while insulin-regulated triglyceride synthesis appears still to be appropriately increased [37]; this apparent paradox has been termed “selective insulin resistance” [38]. However, work using genetic modifications of the insulin-signaling pathway in mice [35, 39] has shown that hepatic DNL, including expression of *Srebf1*, is regulated by insulin; hepatic insulin resistance, as defined by defects in insulin signaling, therefore would prevent insulin from increasing *Srebf1* expression. Furthermore, the hepatic lipid storage and export observed in hepatic insulin resistance may not involve stimulation of DNL by insulin, but may instead reflect packaging of fatty acids coming to the liver from insulin-resistant adipose tissue [40]. In light of these considerations, we utilized insulin’s effect on expression of genes in lipogenic pathways to assess hepatic insulin resistance.

Ad libitum intake of the HF diet suppressed the effects of an oral glucose load, used to raise plasma insulin level, on expression of some of the genes studied (*Srebf1, Fasn, Scd1, Gck, Pck1, and Cpt1a)*. This result may be due, in part, to the high plasma levels of leptin in both HF1 and HF2 rats; leptin has been reported to decrease expression of genes involved in the lipogenic pathway, including *Srebf1* [41] and *Scd1* [42]. In contrast, only the LF, HF1 and HF2 groups appropriately decreased *Insig2* mRNA after the glucose load. Given that Insig2 plays a role in controlling diurnal patterns of metabolism, the disturbance of ad libitum feeding in the VLC, LC and HC, but not LF, HF1 or HF2 groups by feed restriction may have affected regulation of *Insig2* transcription [43].

In Phase 1, even though the HF1 group failed to increase *Srebf1* mRNA in response to the glucose load, expression of *Acaca*, a target of SREBP1c, did increase in both LF and HF1rats. It is possible that SREBP1c may have mediated this response in both groups, since the reduction in *Insig2* mRNA after the glucose load in LF, HF1, and HF2 groups may have led to a decrease in Insig2 protein, thereby increasing conversion of SREBP1c to its mature form. It is also possible that expression of *Acaca* was stimulated by other regulators, such as ChREBP, which is activated by a metabolite of glucose [44]. However, there were no detectable changes in expression of the ChREBP gene (*Mlxipl*) between feed-deprived and 3 h post-glucose states in either LF or HF1 rats.

The observed reduction in lipogenic gene response to the glucose load in the HF1 and HF2 groups is consistent with the decrease in DNL seen in mice on a high-fat diet [45]. In contrast, increased expression of lipogenic genes has been reported in rats [46] and mice [35, 47, 48] consuming high-fat, or high-fat, sucrose-containing diets. However, those findings pertain to gene expression in the feed-deprived state [46, 47] or in a single fed condition, and not a comparison of pre- vs. post-glucose gene expression as in the present study, and so do not assess stimulation of expression by glucose or insulin. Although high cholesterol intakes (4% of diet weight) have been associated with insulin resistance [49] such an effect would not be probable for rats on the HF1, HF2, VLC or LC diets, which contained less than 0.5% cholesterol. Furthermore, mice on 1% cholesterol high-fat diets showed increased expression of SREBP1c [50], not the decrease that we observed.

Weight loss and reduction in plasma leptin levels, regardless of diet, were associated with improved effects of the glucose load on *Fasn, Gck*, and *Pck1* expression in VLC, LC and HC rats (Fig. 4), as compared with the HF1 rats before weight reduction (Fig. 3). However, like the HF2 group, VLC rats did not show significant changes in expression of S*rebf1*, S*cd*, or C*pt1a* in response to the glucose load at the end of Phase 2. This result supports hepatic insulin resistance in VLC rats, despite reduction in hepatic lipid levels and body weights from HF1 levels. The stimulation of *Srebf1* expression by the glucose load in rats on LC or HC diets, which differed in fat and cholesterol levels, suggests that their higher carbohydrate intake, vs. that of VLC rats, had improved their hepatic insulin sensitivity. Regulation of *Srebf1* transcription involves not only insulin, but includes the liver X receptor (LXR), mechanistic target of rapamycin complex (mTorc), and positive feedback from the mature form of SREBP1c [51]. Since dietary protein levels in VLC, LC and HC diets were similar, differences in amino acid intake seem unlikely to account for lower mTorc activation by insulin in the VLC group; similarly high fat intakes of VLC and LC rats also make it unlikely that those groups differed in their intake of dietary regulators of LXR.

Failure of a carbohydrate stimulus to increase *Srebf1* mRNA by rodents consuming high-fat diets has been attributed to their polyunsaturated fatty acid (PUFA) intake [51–53] . This possibility is unlikely to explain the differences between VLC and LC or HC response for the following reasons: 1) PUFA (92% n-6) intakes of VLC (~ 1.57 g/d) and LC (~ 1.25) groups were similar; 2) in other studies, PUFA were consumed near the time of liver sampling, [52–55], whereas rats in our study were feed-deprived for at least 16 h before dissection, precluding an acute effect of dietary PUFA; and 3) if chronically higher PUFA intake were responsible for reducing levels of *Srebf1* mRNA, then lower feed-deprived expression in HF1, HF2, VLC or LC vs. HC groups would be expected, but this was not observed.

Only the HC group showed a significant stimulation of *Scd1* expression in Phase 2; overall expression of *Scd1* was also higher in LF vs. HF1 rats in Phase 1. High carbohydrate diets (e.g., LF and HC) stimulate *Scd1* transcription via SREBP1c and ChREBP [56]. Normal and diabetic mice consuming a diet similar to the LC (18%C, 37%F) showed both reduced insulin sensitivity and lower hepatic expression of *Scd1* than mice consuming a diet similar to the HC or LF diets (63% C, 22%F) [57]; the small difference in their fat intake suggests that the difference in carbohydrate intake was responsible for the effect on *Scd1* expression.

No difference in feed-deprived levels of *Cpt1a* mRNA was observed between any high-fat group (HF2, VLC or LC) and the HC group, although *Cpt1a* mRNA has been reported to be higher in rats on high-fat vs. low-fat diets in the feed-deprived state [58]. However, the finding that only the HC group, but not the HF2, VLC or LC groups, significantly decreased *Cpt1a* mRNA after the glucose load is consistent with the lack of difference in *Cpt1a* expression reported between the fed and feed-deprived state in rats consuming high-fat diets [59]; this lack of suppression of *Cpt1a* expression should promote continued use of fat as a fuel, despite increased availability of glucose.

In Phase 3, the lower responsiveness of hepatic gene expression to the glucose load by the VC3 rats, vs. that of VLC, LC or HC rats at the end of Phase 2, may have been associated with weight loss during the first 3 days after the switch to the HC diet. The stabilization of body weight during days 4–7 on the HC diet by VC7 rats may have promoted better responses. However, VC7 rats still differed from HC rats in their lack of increase in *Scd1* mRNA in response to glucose, and mRNA levels of many genes remained below that exhibited by either VLC or HC rats. These results indicate that full transition from the effects of the VLC to those of the HC diet did not occur within 1 week.

The HC diet promoted greater responses of the insulin-regulated genes, *Srebf*, *Scd1* and *Cpt1a,* to the glucose load than the VLC diet. As discussed above, the higher carbohydrate intake of the HC vs. the VLC or LC groups is likely to have accounted for the greater *Scd1* gene response, and the lower fat intake of the HC vs. the LC or VLC groups may have accounted for the greater *Cpt1a* gene response the glucose load in HC rats. Although the VLC and LC groups had similar fat intakes, only the LC group increased expression of *Srebf* in response to glucose; the severely restricted carbohydrate intake of VLC rats may account for their lack of response.. However, since it is not possible to vary only fat or only carbohydrate intake, while holding protein and energy intake essentially constant, it remains possible that differences in both fat and carbohydrate intake are responsible for the observed dietary effects.

VLC, but not LC rats had elevated HOMA-IR and higher insulin levels after the *ip* glucose load as compared with HC rats, indicating whole body insulin resistance in VLC rats; since LC and VLC groups had similar fat intakes, this finding suggests a mitigating effect of the higher carbohydrate intake of the LC rats. However, the combined effects of high dietary fat and stimulation of expression of lipogenic genes (perhaps including targets of SREBP1c that are involved in esterification) in the LC group may have promoted hepatic TG storage [60]. Nonetheless, hepatic steatosis was not associated with whole body insulin resistance in weight-reduced rats in the present study. In addition, loss of the response of hepatic gene expression to a glucose load was not associated with hepatic steatosis.; Although hepatic lipid levels were similar in HF1, HF2 and LC rats, LC rats showed greater response to the glucose load than HF1 or HF2 rats, or than the VLC group which had lower hepatic lipid levels.

In HC rats, the insulin-stimulated increases in hepatic SREBP1c and its targets would be expected to promote conversion of glucose to fatty acids and triglyceride; decreases in hepatic Cpt1a would be expected to suppress fat oxidation in favor of glucose oxidation. Both processes should support the ability of a given concentration of insulin to lower plasma glucose levels after a glucose load; this is consistent with lower whole body insulin resistance in HC rats. Lack of this hepatic response in VLC rats would be expected to diminish the glucose-lowering effect of insulin; this is consistent with the higher insulin levels observed for the same level of glucose in VLC vs. HC rats.

## Conclusion

The carbohydrate and fat contents of diets producing the same weight loss differentially affected body composition and insulin resistance in obese rats. The VLC diet promoted whole body insulin resistance and diminished the effect of a glucose load on hepatic expression of some insulin-regulated genes, as compared with HC or LC diets. These findings reveal adaptations produced by a VLC weight loss diet that could promote hepatic insulin resistance, thereby lessening the effect of insulin on metabolic pathways.

## Abbreviations

ChREBP: Carbohydrate Regulatory Element Binding Protein
GTT: Glucose tolerance test
HC: High-carbohydrate
HF1: High-fat Phase 1
HF2: High-fat Phase 2
ip: Intraperitoneal
IR: Insulin resistance
LC: Low-carbohydrate
LF: Lower fat
MUFA: Monounsaturated fatty acid
PUFA: Polyunsaturated fatty acid
SREBP1c: Sterol regulatory element binding protein 1c
TG: Triglyceride
VLC: Very low-carbohydrate

## The following gene names

*Acaca*: *A*cetyl-CoA carboxylase
Cpt1a: Carnitine Palmitoyl transferase-1a
*Fasn*: Fatty acid synthase
Gck: Glucokinase
*Insig2*: Insulin signaling protein-2
*Pck1*: Phosphoenolpyruvate carboxykinase-1
*Scd1*: Stearoyl-CoA desaturase-1
*Srebf1*: Sterol regulatory element-binding protein 1
